# A Novel Method for Notable Reducing Phase Transition Temperature of VO_2_ Films for Smart Energy Efficient Windows

**DOI:** 10.3390/nano10010058

**Published:** 2019-12-25

**Authors:** Huan Guan, Dongping Zhang, Yu Yang, Yi Liu, Aihua Zhong, Qicong He, Jiahua Qi, Ping Fan

**Affiliations:** Shenzhen Key Laboratory of Advanced Thin Films and Applications, College of Physics and Optoelectronic Engineering, Shenzhen University, Shenzhen 518060, Chinaliuy@szu.edu.cn (Y.L.); zhongah@szu.edu.cn (A.Z.); 1800211011@email.szu.edu.cn (Q.H.); qijiahua883@163.com (J.Q.); fanping@szu.edu.cn (P.F.)

**Keywords:** vanadium dioxide, phase transition temperature, ultra-thin heavy Cr-doped layer

## Abstract

Although Vanadium dioxide (VO_2_) has a potential application value for smart energy efficient windows because of its unique phase transition characteristic, there are still many obstacles that need to be overcome. One challenge is to reduce its high transition temperature (***ζ****_c_* = 68 °C) to near room temperature without causing its phase transition performance degradation. In this paper, a novel method was employed that covered a 3 nm ultra-thin heavy Cr-doped VO_2_ layer on the pure VO_2_ films. Compared with the as-grown pure VO_2_, obviously, phase transition temperature decreasing from 59.5 °C to 48.0 °C was observed. Different from previous doping techniques, almost no phase transition performance weakening occurred. Based on the microstructure and electrical parameters measurement results, the mechanism of ***ζ****_c_* reducing was discussed. The upper ultra-thin heavy Cr-doped layer may act as the induced role of phase transition. With temperature increasing, carrier concentration increased from the upper heavy Cr-doped layer to the bottom pure VO_2_ layer by diffusion, and induced the carrier concentration reach to phase transition critical value from top to bottom gradually. The present method is not only a simpler technique, but also avoids expensive alloy targets.

## 1. Introduction

Vanadium dioxide (VO_2_) is one of the most interesting smart materials for its reversible metal-insulator transition (MIT) near room temperature (***ζ****_c_* = 68 °C) [1], in which optical, electrical, and other physical properties (transmittance, reflectance, emittance, refractive index, electrical resistivity etc.) [2,3] will be sharp changed by external stimuli (applied field or voltage [4], incident light [5], temperature variation [6], mechanical stress [7], pressure [8], etc.) in the transition process. These unique properties make VO_2_ a suitable candidate for various technological potential applications in many fields, such as tunable filters [9], switching devices [10], memory materials [11], laser protection [12], ultrafast sensors [13], and Mott transistors [14]. Great attention has been attracted recently for the material both in scientific and technological applications. 

In the last years, an increasing number of researches have been published and discussed VO_2_-based applications. Among all the VO_2_ application researches, the most attractive one is the smart energy efficient window application. Due to it exhibiting a reversible transformation from an infrared (IR)-transparent at low temperature semiconductor phase to an IR-reflective at high temperature metallic phase, meanwhile maintaining visible transmittance, VO_2_ is the most promising material for smart energy efficient windows [15]. Although lots of works have been done over the past years, still many obstacles should be overcome for VO_2_-based smart window real applications. One challenge is to reduce the transition temperature ***ζ****_c_* of VO_2_ from 68 °C to around room temperature [16]. Previous reports showed that the ***ζ****_c_* of VO_2_ could be reduced by a range of technologies, such as elemental doping [17], introducing stress [18] and defects [19], and chemical stoichiometry adjustment [20]. Doping is considered to the most effective strategy to reduce the ***ζ****_c_* of VO_2_, especially for high-valence ions doping (such as Nb^5+^, Mo^6+^, W^6+^, Tb^3+^, etc.) [21,22,23,24]. Chae et al. [25] reported that the doped VO_2_ with W and Ti using the sol-gel method led to a change of transition temperature and reduces the properties of MIT. M. Panagopoulou et al. [26] found that Mg doping could reduce the transition temperature of VO_2_, and the 6 °C/at % Mg-doped VO_2_ thin film on ZnO substrate presented the lower ***ζ****_c_* around 35 °C, but the energy modulation had sharply decreased. Jin et al. [27] reported that doping W into VO_2_ by using high-energy ion implantation appeared to have a much higher ***ζ***_c_ reduction efficiency compared with other methods. Carlos Batista et al. [28] reported that the ***ζ****_c_* would decrease 8 °C/at % by Nb doping. Most of previous works’ results exhibited that the phase transition performance as well as solar energy modulation capability of VO_2_ films weakened after doping, though the sharp decreasing of ***ζ***_c_ was obtained.

In this work, pure VO_2_ films were prepared by reactive pulsed magnetron sputtering, and a 3 nm ultra-thin heavy Cr-doped VO_2_ layer was employed to cover on the prepared pure VO_2_ film surface. By annealing in high vacuum environment at a temperature of 250 °C, notable phase transition temperature *ζ_c_* reduction of VO_2_ films was observed, while phase transition performance of the samples has hardly weakened. The present method is not only a simpler technique, but also avoidsexpensive alloy targets.

## 2. Experimental Details

**Figure 1:**
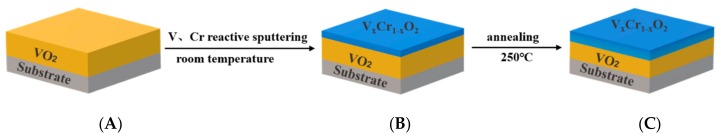
Schematic of the films structure, (**A**) is the pure VO_2_ thin film, (**B**) is the unannealed Cr-doped VO_2_ thin film, (**C**) is the Cr-doped VO_2_ thin films annealing at 250 °C.

VO_2_ thin films were prepared on K_9_ glass substrates at 440 °C by reactive pulsed direct current magnetron sputtering in vacuum chamber with turbo molecular bump system. High purity (99.99%) vanadium was used as target with diameter of 100 mm. First, the vacuum chamber was evacuated to a base pressure of 1.0 × 10^−3^ Pa before deposition. Then Ar (99.99%) and O_2_ (99.99%) gases were led into chamber with flow rate of 40 sccm and 1.5 sccm acted as working gas and reactive gas, respectively. Before films’ deposition, 10 minutes pre-sputtering was performed in Ar atmosphere to remove surface contaminant and oxide layer on the target in order to maintain deposition process stability and obtain high purity VO_2_ films. A pulsed direct current supply (Advanced Energy Industries) was used for films magnetron sputtering deposition. The 50 nm thick VO_2_ thin films were prepared under working pressure of 0.6 Pa. A 3 nm ultra-thin heavy Cr-doped VO_2_ layer was deposited on the top of pure VO_2_ films by direct current reactive co-sputtering method at room temperature with power of 10 W, and films’ thickness was controlled by deposition time. Subsequently, the samples were annealed in high vacuum atmosphere (4.0 × 10^−4^ Pa) at 250 °C for 30 min. The schematic of thin films’ preparation process is shown in Figure 1, and the samples are labeled as A, B, and C, respectively.

The transmittance thermal-hysteresis curves at λ = 1550 nm of the prepared samples were measured with a thin film phase transition measurement system (PERFECT PTM-1700, Perfect Opto-electronics Technology, Shenzhen, China), and the temperature ranged from room temperature to 90 °C. Microstructure of the films was identified by X-ray diffraction (XRD) in θ–2θ coupled scanning mode (Ultima IV, Rigaku, Tokyo, Japan) with Cu Κ_α_ radiation, which the wavelength is 0.15406 nm. The diffraction angle ranged from 10° to 80° with step by 0.02°. The vibrational modes in VO_2_ films were examined by using Raman Microscopy (inVia, Renishaw, Gloucestershire, England) with a 532 nm wavelength laser as excitation source, and the laser power was kept as low as 2 mW in order to minimize additional heating effects, and the wave number range varied from 100 to 800 cm^−1^. The transmittance of the thin films at room temperature and 80 °C were obtained using a spectrophotometer (UV-3600 PLUS, SHIMADZU, Shimadzu, Japan) in the range of 300–2500 nm. The samples surface morphologies were determined by field emission scanning electron microscopy (SEM). The temperature-dependent sheet-resistance variation of films was characterized using a four-point probe system, and the thin film temperature was ramped up from room temperature to 90 °C at a step of 1 °C in measurement process. The carrier concentration of the samples was measured by using the Hall Effect Measurement System (Bio Rad, HL5500 PC, Nanometrics, Milpitas, California, USA), and the entire sample was heated from 35 °C to 80 °C.

## 3. Result and Discussion

**Figure 2:**
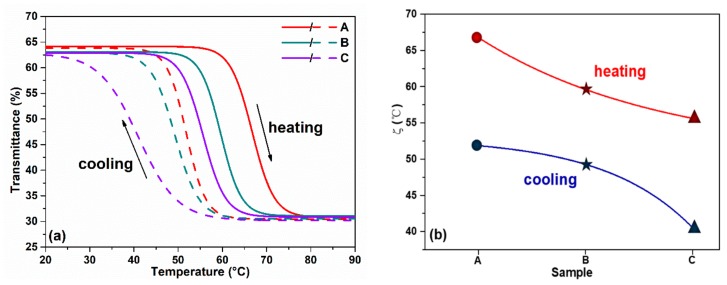
(**a**) Transmittance thermal hysteresis loop of all samples at λ = 1550 nm, (**b**) the *ζ_ch_* and *ζ_cl_* of all samples.

The thermochromism characteristics of VO_2_ films were investigated by measuring their temperature dependence of IR transmittance at λ = 1550 nm. The transmittance-temperature dependence curves in the heating and cooling processes are shown in Figure 2a, and the transition temperature in the heating process ***ζ****_ch_* and cooling process ***ζ****_cl_* are shown in Figure 2b. The thermal hysteresis loop curves clearly exhibit that all samples have high phase transition performance. The transmittance at *λ* = 1550 nm changed from 65% in low temperature semiconductor state to 30% in high temperature metallic state. For sample A of the as-grown pure VO_2_, the heating and cooling phase transition temperatures are ***ζ****_ch_* = 67 °C and ***ζ****_cl_* = 52 °C, respectively. The average phase transition temperature ***ζ****_c_ =* (***ζ****_ch_ +* ***ζ****_cl_*)*/*2 of sample A is 59.5 °C. When the sample surface was covered with an ultra-thin heavy Cr-doped layer, notable reducing of phase transition temperature was found, either unannealed or annealed. For the unannealed sample B, the ***ζ****_c_* decreasing may be related to the interface stresses between the two layers. While for sample C, phase transition temperature further decreased from 54.5 °C to 48.0 °C after annealing. Figure 2a also shows that the phase transition amplitude almost keeps constant among the different samples.

**Figure 3:**
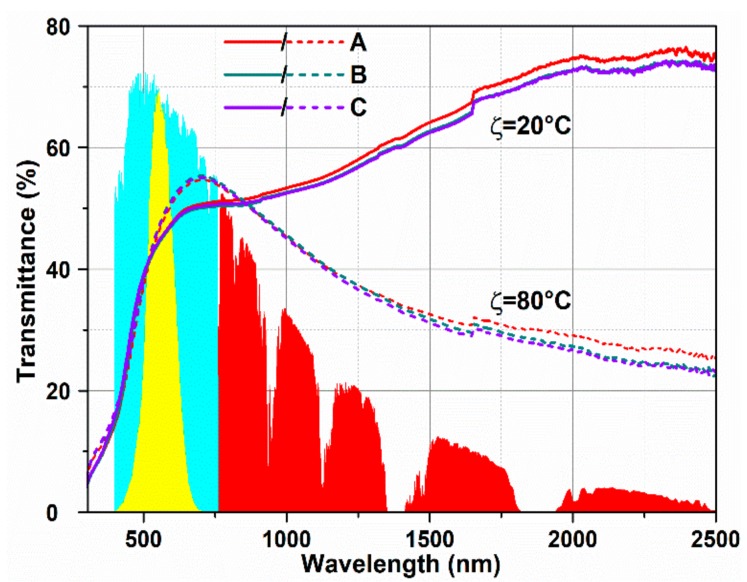
Optical transmittance spectra (300–2500 nm) at room temperature and 80 °C of the samples. The colored areas indicate the normalized values of spectral irradiance corresponding to the visible (blue) and near infrared radiation (red) ranges of solar spectra, and the yellow area indicates the values of eye sensitivity function.

Figure 3 shows the transmittance spectra ranged from near ultraviolet to near-infrared region of the samples at ambient temperature and 80 °C, respectively. All the films show transmittance changes in the IR region after MIT. We also noticed that the transmittance spectra of the three samples are almost overlapped. Only a weak change of transmittance spectra was observed after being covered ultra-thin heavy Cr-doped layer. Combining the results of the above transmittance thermal hysteresis loop, we can conclude that the overlayer has not induced weakening of the phase transition performance. Based on the transmittance spectra, solar energy modulation ability of the samples was calculated according to the formula below [29]:

(1) Ti=∫ϕiλTλdλ∫ϕiλdλ

(2) $${\;\Delta T}_{\mathrm{IR},\mathrm{sol}}\;=\;T_{\mathrm{IR},\mathrm{sol}}\left(20\;{}^{\circ}C\right)-T_{\mathrm{IR},\mathrm{sol}}\left(80\;{}^{\circ}C\right).$$

where *T*(*λ*) is the transmittance at wavelength *λ*, *i* denotes lum or sol, *ϕ_sol_*(*λ*) is the solar irradiance spectrum at air mass 1.5, which likes the angle of incidence of sunlight is 37°, and *ϕ_lum_*(*λ*) is the standard luminous efficiency function for the photopic vision of human eyes (380–780 nm). *T_sol_* is effective utilization of solar energy in thin films, and ΔT_IR,sol_ is the solar energy modulation of VO_2_ films in IR region, the region of 760–2500 nm is always chosen for calculation. According to the above formulas, the three samples have almost the same solar energy modulation capability of ΔT_IR,sol_ = 12%, which meets the requirement of the smart windows application.

**Figure 4:**
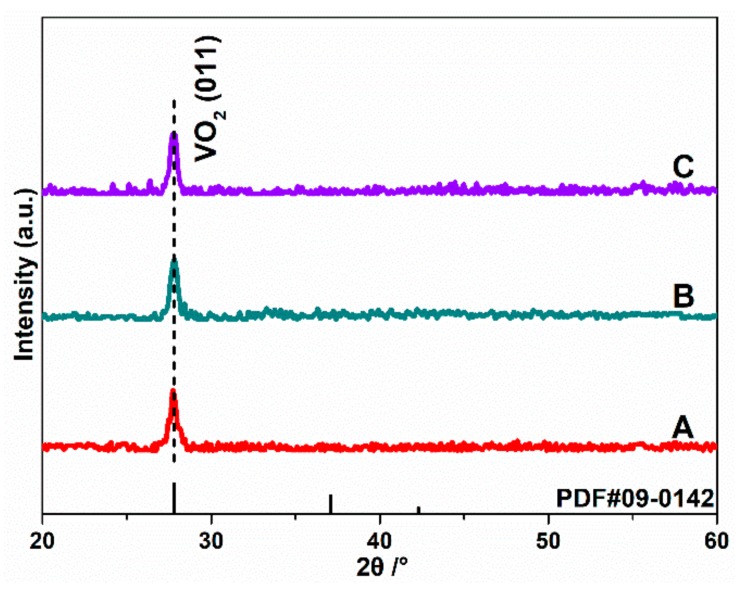
XRD patterns of all samples. (A) is the pure VO_2_ thin film, (B) is the unannealed Cr-doped VO_2_ thin film, (C) is the Cr-doped VO_2_ thin films annealing at 250 °C.

Figure 4 shows the XRD spectra of the three different samples prepared with different procedures. The standard pattern of monoclinic (M) VO_2_ (PDF#09-0142) is also present together for reference. In the XRD patterns, the peaks located at about *2*θ = 27.76° are corresponding to the characteristic pattern of VO_2_, which shows the structure of monoclinic type with a (011) preferred orientation. The characteristic peaks of chromium oxides are not observed in sample B and C, indicating that chromium oxides in covered layer is too thin to detect, or Cr atoms entered the crystal lattice of VO_2_ rather than a separate phase. We notice that the (011) diffraction peak position of 27.76° in our study lower than 27.86° of VO_2_ powder from standard powder diffraction file (PDF) card, the residual stress may play the key role to the diffraction peak blue shifting.

**Figure 5:**
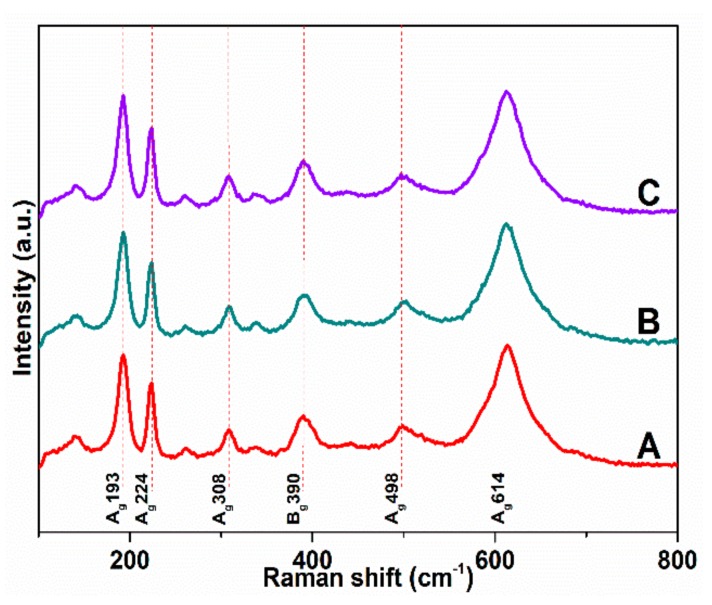
Raman spectra of all samples. (A) is the pure VO_2_ thin film, (B) is the unannealed Cr-doped VO_2_ thin film, (C) is the Cr-doped VO_2_ thin films annealing at 250 °C.

Raman spectra of the VO_2_ films at room temperature is shown in Figure 5. The spectra reveal that all samples have the same characteristic peaks, which appear at 193 cm^−1^, 224 cm^−1^, 308 cm^−1^, 390 cm^−1^, 498 cm^−1^, and 614 cm^−1^, identified to the VO_2_ monoclinic phase [30]. No characteristic peaks of chromium oxides or other vanadium oxides appear, indicating that the main ingredient of films is VO_2_, which can explain the temperature-transmittance hysteresis loop. The monoclinic phase is thus characterized by 18 Raman active modes with 9A_g_ and 9B_g_ modes [31]. Among 193 cm^−1^, 224 cm^−1^, 308 cm^−1^, 498 cm^−1^, and 614 cm^−1^ confirm the A_g_ symmetry mode, another 390 cm^−1^ corresponds to the B_g_ phonon mode [32]. This result is consistent with previous reports. The peaks at 193 cm^−1^, 224 cm^−1^ are assigned to V-V vibration modes, whereas those in the high frequency of 308 cm^−1^, 498 cm^−1^, and 614 cm^−1^ are assigned to V-O vibration modes [33].

**Figure 6:**
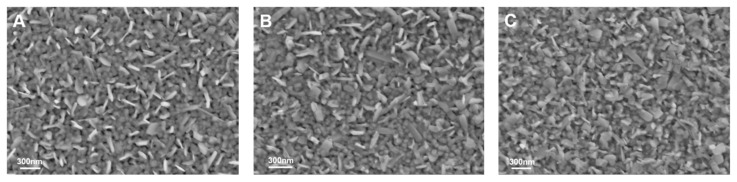
SEM images of as-grown pure VO_2_ and an ultra-thin Cr-doped VO_2_ layer. (**A**) is the pure VO_2_ thin film, (**B**) is the unannealed Cr-doped VO_2_ thin film, (**C**) is the Cr-doped VO_2_ thin films annealing at 250 °C.

Figure 6 shows the surface morphologies of the VO_2_ film samples. All samples exhibit high crystallinity, continuous and dense structure, which are consistent with XRD results. For as-grown pure VO_2_ sample A, some nanorods discontinuous distributed on the surface. While for the sample C, which covered with an ultra-thin heavy Cr-doped layer and 30 min vacuum atmosphere annealing, these nanorods almost disappeared. Due to the nanorods still could be seen on the surface of unannealed sample B, it is reasonable to deduce that the nanorods as well as ultra-thin covered layer has been completely integrated into the film matrix in sample C.

**Figure 7:**
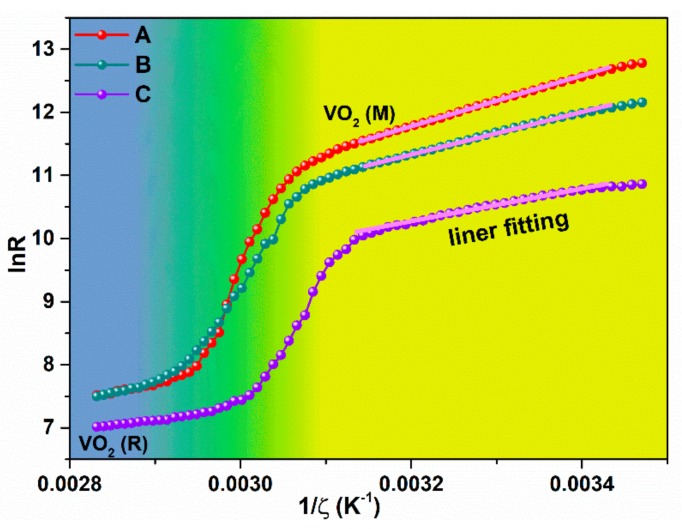
The *lnR–1/ζ* curve of all samples; the activation energy in the semiconductor phase is determined by linearly fitting the curve in low temperature state. (A) is the pure VO_2_ thin film, (B) is the unannealed Cr-doped VO_2_ thin film, (C) is the Cr-doped VO_2_ thin films annealing at 250 °C.

Temperature dependence of the electrical resistivity *R* curves of VO_2_ films in heating process are shown in Figure 7. Abruptly change of resistance from low temperature to high temperature has been seen like IR-transmittance-temperature curve. We also noticed that the sheet resistance decreased in room temperature state after covered with heavy Cr-doped layer either unannealed or annealed.

The temperature dependence of electrical resistivity of VO_2_ thin films in the semiconducting state can be expressed as [34]:

(3) $$R=R_{0}\exp\left(\frac{E_{c}-E_{f}}{k\zeta }\right)$$

where *E_c_* is the energy of the edge of the conduction band, *E_f_* is the Fermi level, *k* is the Boltzmann constant, and *R_0_* is the resistance at ***ζ***→∞.

Temperature coefficient of resistance (TCR) defined as the slope of the natural logarithm of electrical resistivity *R* in semiconducting state with temperature [35]:

(4) $$TCR=\frac{1}{R}\frac{dR}{d\zeta }$$

Combining Equations (3) and (4) yields [36]

(5) $$\Delta E=-k\zeta^{2}\times TCR$$

This relation links the activation energy Δ*E = E_c_* − *E_f_* to TCR [37]. The activation energy in semiconductor state and conductivity at room temperature in our work are calculated (see Table 1). In ambient temperature, the carrier concentration of the three samples are 3.0163 × 10^17^ cm^−3^, −6.8935 × 10^17^ cm^−3^, and −1.9045 × 10^18^ cm^−3^, respectively. It is clear that the VO_2_ samples with heavy Cr-doped ultra-thin layer have higher conductivity and carrier concentration compared with the as-grown sample A. Due to the low conductivity in pure VO_2_ bottom layer, it reveals that higher concentration carriers may only locate within the upper layer locally.

**Figure 8:**
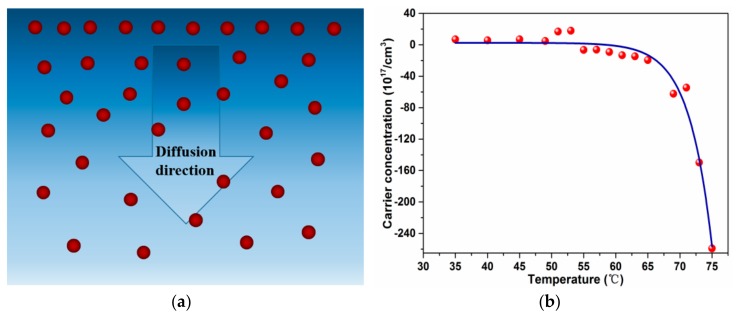
(**a**) Schematic of carrier distribution and diffusion in the cross-section of VO_2_ thin films. (**b**) Temperature-dependent carrier concentration of pure VO_2_ thin films.

The transition temperature can be affected by lattice strain [38], crystal size [39], ions doping [40], etc. From XRD spectra and SEM images, it can be seen that the diffraction peak position and grain size are basically unchanged in this work. So the phase transition temperature variation attributed to lattice strain and grain size is negligible in our research. It is reported that the phase transition behavior of the VO_2_ thin films also could be influenced by carrier concentration. C. W. Zou [41] reported that the MIT could be controlled by modifying carrier density. For the Mott phase transition, the critical electron density *n_c_* can be expressed as below equation:

(6) $$n_{c}={\left(\frac{m^{*}e^{2}}{4\hbar^{2}\varepsilon }\right)}^{3}$$

where *ε* is the dielectric constant (∼100 for VO_2_), *ħ* is the Planck constant, and *m*^∗^ and *e* are the effective mass and charge of an electron, respectively [42] For MIT in VO_2_, *n_c_* ≈ 3 × 10^18^ cm^−3^ [43]. For sample B and C with low activation energy, the samples’ surface is heavy Cr-doped layer and has high carrier concentration. It is easier to generate more carriers to reach the phase transition critical value of *n_c_* and first induce local phase transition. MIT process will lead to carrier concentration increasing further. The carriers will diffuse from upper layer to bottom according to Fick’s first law:

(7) $$J=-D\frac{dn}{dx}$$

where *D* is carrier diffusion coefficient, *J* is diffusion flux, and *dn/dx* is the carrier concentration gradient [44]. With temperature increasing, the diffusion coefficient *D* and carrier concentration gradient *dn/dx* in the vertical direction of the film surface increased too. It will lead to higher carrier diffusion flux and phase transition occurring from upper layer to bottom layer gradually. It is reasonable to deduce that the upper heavy Cr-doped ultra-thin VO_2_ film play the role of phase transition induction layer initially, which could reduce the phase transition temperature ***ζ****_c_* due to the carrier injection (Figure 8a).

Figure 8b shows the temperature-dependent carrier concentration of pure VO_2_ thin films. In the low-temperature insulating monoclinic state, the free-carriers type is holes. With temperature increasing, carriers gradually changed to electrons, and a sharp increasing of electrons concentration was observed when temperature increase to ~70 °C, and the film turns into the metal rutile state.

## 4. Conclusions

In summary, VO_2_ films were prepared by reactive pulsed magnetron sputtering, and a 3 nm ultra-thin heavy Cr-doped VO_2_ layer prepared by reactive co-sputtering was employed to cover the pure VO_2_ film. Then the covered samples were annealed in vacuum atmosphere. By characterizing the phase transition performances, microstructures, and electrical properties of the samples, phase transition temperature sharp decreasing of the covered ultra-thin heavy Cr-doped layer films was observed, and annealing could strengthen this effect. No obvious change of the microstructure and phase transition performance has been found before and after covering an ultra-thin layer on the pure VO_2_ film. The three samples have almost the same solar energy modulation capability of ΔT_IR,sol_ = 12%. It is reasonable to deduce that the upper ultra-thin high Cr-doped layer, which has high carrier concentration, may act as induced-layer to generate more carriers to reach the threshold *n_c_* and induced phase transition. This process will occur from upper layer to bottom layer gradually until phase transition occurs throughout the VO_2_ film by carrier diffusion with temperature increasing. This is a simple method to reduce ***ζ****_c_* and keep phase transition phase performance.

## Figures and Tables

**Table 1:** Activation energy in semiconductor state and conductivity at room temperature of all samples.

Sample	A	B	C
activation energy Δ*E* (eV)	0.33	0.27	0.23
conductivity *σ* (S/m^2^)	12.95	24.10	86.70
carrier concentration *n* (cm^−3^)	3.0163 × 10^17^	−6.8935 × 10^17^	−1.9045 × 10^18^
